# Raids on the Central Park Menagerie

**Published:** 1881-01

**Authors:** W. A. Conklin

**Affiliations:** Director C. P. Menagerie; New York


EDITOR OF JOURNAL OF COMPARATIVE MEDICINE:—

On the night of November 25, a pack of dogs entered the park, and killed fourteen black swans, together with other water fowl. These raids by dogs are of frequent occurrence, and it is almost impossible to guard against them. When the deer were enclosed in the upper part of the park, the annoyance from these marauders was very great. Their plan of operation was, usually, to have a small dog creep through the wire fence, start the deer, which, when frightened, would either jump over the fence, or, by dashing against it, break a panel, escape, and become victims to the larger dogs awaiting outside. In these raids, the dogs seem to kill for mischief clearly, as they never eat the flesh. Dr. Hadden, in his article on the castration of the dog, for the protection of sheep and smaller animals and fowl, published in the second number of the ARCHIVES, gives very forcible arguments in favor of legislation in this matter, and the recent destruction of valuable fowl is an additional argument, proving the necessity of adopting some such measure of protection; and I will here confirm what he has set forth in his paper, that the male canine are the only offenders, and that they are always in company, or packs, in these raids.

W. A. CONKLIN,

*Director C. P. Menagerie.*

NEW YORK, Dec. 14, 1880.

